# First report of a DIM-1-producing *Pseudomonas aeruginosa* in Spain: genomic characterization and resistance determinants

**DOI:** 10.1128/aac.01311-25

**Published:** 2025-11-18

**Authors:** Irene Cadenas-Jiménez, Aida González-Díaz, Dàmaris Berbel, Rosa Costa-Primo, Guillem López de Egea, Fe Tubau, M. Angeles Domínguez, Carmen Ardanuy, Jordi Cámara, Sara Marti

**Affiliations:** 1 Microbiology Department, Hospital Universitari Bellvitge, IDIBELL-UB, Barcelona, Spain; 2 Research Network for Respiratory Diseases (CIBERES), ISCIII 38176, Madrid, Spain; 3 Department of Pathology and Experimental Therapeutics, University of Barcelona 16724, Barcelona, Spain; 4 Research Network for Infectious Diseases (CIBERINFEC), ISCIII 38176, Madrid, Spain; Shionogi Inc., Florham Park, New Jersey, USA

**Keywords:** beta-lactamase, *Pseudomonas aeruginosa*, integron, genomic island, antimicrobial resistance

## Abstract

This study reports the first DIM-1-producing *Pseudomonas aeruginosa* identified in Spain. The multidrug-resistant ST233 clone harbored the *bla*_DIM-1_ gene within the class 1 integron In1592, together with aminoglycoside resistance genes *aph(3'*) and *aphA*. This mobilizable element is a fragment of the PAGI-18 genomic island previously identified in Poland, but with different flanking insertion sequences, suggesting independent acquisition events. These findings highlight the importance of genomic surveillance of rare resistance determinants and mobile genetic elements.

## INTRODUCTION

Carbapenem resistance among Gram-negative bacteria is a global health concern, driven by the rise of acquired resistance mechanisms through horizontal gene transfer and point mutations (1). Among these, metallo-β-lactamases (MBLs) represent a significant threat due to their ability to hydrolyze nearly all β-lactams, including carbapenems, and their resistance to most serine β-lactamase inhibitors. MBLs are frequently encoded on mobile genetic elements, which facilitates their rapid spread across different bacterial species and geographic regions (2). Several types of MBLs have been described, with IMP, NDM, and VIM being the most widely distributed. The genes encoding these enzymes are often located within class 1 integrons, which promote their mobilization and dissemination among bacteria, such as *Enterobacterales*, *Pseudomonas aeruginosa*, and *Acinetobacter* spp. (3).

DIM-1 is one of the most recently identified MBLs. This β-lactamase was first detected in a *Pseudomonas stutzeri* isolate recovered from a Dutch patient after tibial osteomyelitis surgery. The gene was embedded within a two-gene cassette, alongside *aadB*, within a class 1 integron located on a 70 Kb plasmid (4). Since then, this enzyme has remained rare globally, with sporadic cases reported mainly from Asian countries. Only two *P. stutzeri* isolates harboring DIM-1 have been reported to date (Table 1): the original Dutch strain isolated in 2007, and a second, retrospectively identified from India, dating back to 2000. These isolates were genetically distinct, suggesting independent acquisition events (5).

**TABLE 1:** Overview of all β-lactamase DIM-1 identified in *Pseudomonas* spp.^*a*^

Isolation year	Bacterial species	Strain	Country	Sample/type of infection	MLST	Co-resistance	Resistance genes^*b*^	Accession number	Reference
2007	*P. stutzeri*	13	Netherlands	Tibial osteomyelitis	ND	PIT, CTZ, CEP, IMI, CIP, GEN, TOB	*bla*_DIM-1_, *aadB*	GU323019	(4)
2000	*P. stutzeri*	10627	India	ND	ND	PIT, CTZ, CIP, GEN	*bla*_DIM-1_, *aadB*	ND	(5)
2016	*P. aeruginosa*	PA27	India	Pus	ND	CTX, CEP, MER, CIP, AMI, GEN,	*bla*_DIM-1_, *aadB*	ND	(1)
2014	*P. aeruginosa*	1334/14	Poland	Endophthalmitis	ST234	PIT, CTZ, CEP, CTT, AZT, IMI, MER, CIP, AMK, GEN, TOB	*bla*_NDM-1_, *bla*_PME-1_, *bla*_DIM-1_, *bla*_OXA-10_, *aph(3')Ib*, *aph(3')IIb*, *aph(3')-VI*, *strAB*, *aadA1*, *aadB*, *aacC3*	CP035739	(6)
2016/17	*P. aeruginosa*	194, 195, 202, 221, 277, 279, 280, 428, 452, 514, 522, 591	Myanmar	Wound, blood, urine	ST1047	CTZ, CEP, AZT, IMI, MEM, CIP, AMK	*bla*_NDM-1_, *bla*_DIM-1_		DRA007442		(7)
2012/13	*P. aeruginosa*	82, 36	Vietnam	Urethral stenosis, pneumonia	ST3440 ST1420	CTZ, AZT, IMI, CIP, GEN	*bla*_NDM-1_, *bla*_IMP-15_	ND	(8)
2015	*P. aeruginosa*	97, 130, 140, 142	Ghana	Urine, wound, peritoneal fluid	ST234	PIT, CTZ, CTT, CTV, AZT, IMI, MER, CIP, AMK, GEN, TOB	*bla*_DIM-1_, *bla*_IMP-1_, *bla*_OXA-10_, *bla*_OXA-129_, *aadA1*, *aph(3')-IIb*, *aacA4*, *aadA6*	PRJNA411997	(9)
2019	*P. aeruginosa*	BJ86	China	Sputum	ST2446	PIT, CTZ, CEP, IMI, MER, CIP, GEN, TOB	*bla*_DIM-1_, *bla*_OXA-4_, *aac(6')-Ib*, *aac(6')-IIa*	NMDC60134482	(10)
2022/23	*P. aeruginosa*	SOM_OS017M	Tanzania	Venous leg ulcer	ST2305	PIT, CTZ, IMI, CIP, GEN	*bla*_DIM-1_, *bla*_OXA-10_, *bla*_OXA-395_, *ant(2'')-Ia*, *ant(3'')Ia*, *aph(3')-IIb*	PRJEB8148	(11)
2023	*P. aeruginosa*	HUB-PA33644	Spain	Pyelonephritis	ST233	PIT, CTZ, CEP, CTT, CTV, IMI, MER, CIP, AMK, TOB	*bla*_DIM-1_, *bla*_OXA-4_, *bla*_OXA-486_, *aadA2*, *aph(3')-Ib*, *aph(3')-IIb*, *aph(3')-VI*	PRJEB96242	This study

^*a*^ AMI, amikacin; AZT, aztreonam; CEP, cefepime; CTX, cefotaxime; CTZ, ceftazidime; CTV, ceftazidime/avivactam; CTT, ceftolozane/tazobactam; CIP, ciprofloxacin; GEN, gentamycin; IMI, imipenem; MER, meropenem; PIT, piperacillin/tazobactam; TOB, tobramycin; ND, not determined.

^*b*^ Acquired genes associated with beta-lactam and aminoglycoside resistance.

Following these initial identifications, the *bla*_DIM-1_ gene has been detected in *P. aeruginosa* strains, although such reports also remain limited and geographically dispersed. The first isolate was reported in India in 2018 (1), followed by a Polish strain belonging to ST234 reported in 2019 that co-produced NDM-1 (6). Between 2016 and 2017, a cluster of 12 ST1047 DIM-1-producing *P. aeruginosa* isolates was detected in Myanmar (7). Other reports include two isolates from Vietnam (ST3440 and ST1420), both producing DIM-1 and IMP-15 (8), as well as four ST234 isolates reported from Ghana (2021) carrying DIM-1 and IMP-1 (9). More recently, two additional *P. aeruginosa* isolates have been identified in China (ST2446) (10) and Tanzania (ST2305) (11). Notably, the Chinese isolate carried the DIM-1 on a 522 Kb megaplasmid (pBJ86) that contained a multiresistance region with 18 different antimicrobial resistance genes (10).

Here, we report the first DIM-1-producing *P. aeruginosa* in Spain, and the second documented case in Europe. The strain was isolated from a urine sample of a 23-year-old male patient admitted to Bellvitge University Hospital (HUB), a tertiary hospital in southern Barcelona (Spain). The patient had arrived from Honduras only 2 months earlier, suggesting a potentially imported case. His medical history included bilateral ureteral obstruction, managed with long-term double-J stents and a suprapubic catheter due to bladder dysfunction and recurrent hydronephrosis. Prior to admission to HUB, he had received multiple courses of broad-spectrum antibiotics due to previous bacterial infections. In August 2023, the patient was admitted to our hospital with fever, chills, dysuria, and acute kidney injury, symptoms consistent with obstructive pyelonephritis. Urine cultures revealed the presence of *P. aeruginosa* and *Proteus mirabilis,* and empirical treatment with ertapenem was initiated. Once antimicrobial susceptibility testing results were available and *P. aeruginosa* was confirmed to have an XDR phenotype, the treatment was adjusted to a combination of ertapenem and aztreonam.

The *P. aeruginosa* HUB-PA33644 strain was characterized both phenotypically and genotypically. Antimicrobial susceptibility was tested by microdilution using commercially available panels (NM63, MicroScan Walkaway system, Beckman Coulter). Additionally, cefiderocol susceptibility was tested by e-test (Liofilchem), following the clinical breakpoints recommended by the European Committee on Antimicrobial Susceptibility Testing (www.eucast.org). Whole-genome sequencing was conducted using the SMRTbell prep kit on a Sequel II system (PacBio, USA) following the manufacturer’s instructions. Sequence assembly was performed from 6,873,728 total bases (raw sequencing data available in European Nucleotide Archive under the accession code PRJEB96242), with a read depth of 130 across the genome and read length N50 of 12,796 bases, using the SMRT Link v8.0.0 interface and Microbial Assembly analysis application (Pacific Biosciences). The ResFinder database was used to identify resistance genes, and Geneious R9 software (version 9.1.7, Biomatters) was used to analyze the genomic surroundings of the DIM-1 β-lactamase.

Molecular typing classified the strain as ST233, a high-risk clone common in Europe and frequently associated with the acquisition of the MBL VIM-2 (12). Notably, most of the *P. aeruginosa* isolates carrying the *bla*_DIM-1_ gene, including the present strain, belong to distinct sequence types, suggesting multiple independent acquisition events for this resistance determinant rather than clonal dissemination. Phenotypically, the strain displayed an multi-drug resistant (MDR) profile, being resistant to cephalosporins, carbapenems, ciprofloxacin, and amikacin (Table 2). Genomic analysis revealed several resistance determinants. The aminoglycoside-modifying enzyme APH(3′)-VI was linked to amikacin resistance while preserving susceptibility to tobramycin. The acquired β-lactamase *bla*_OXA-4_ (OXA-1 family), along with the intrinsic AmpC (*bla*_PDC3_) and OXA-50 family (*bla*_OXA-486_), mediated resistance to penicillins and cephalosporins. These were complemented by the presence of the MBL DIM-1, which conferred resistance to broad-spectrum cephalosporins and carbapenems, including their combinations with β-lactamase inhibitors, such as ceftazidime/avibactam and ceftolozane/tazobactam. In contrast, cefiderocol remained active against this strain. Additionally, mutations in *gyrA* (T83I, D87Y) and *parC* (S87L) were associated with resistance to ciprofloxacin.

**TABLE 2:** Phenotypic and genotypic characterization of *P. aeruginosa* strain HUB-PA33644^*a*^

Feature	Results
Sequence type (ST)	233
Serotype	O6
Antimicrobial agent	
Piperacillin-tazobactam	>64 mg/L (R)
Ceftazidime	>16 mg/L (R)
Cefepime	>16 mg/L (R)
Ceftolozane/tazobactam	>256 mg/L (R)
Ceftazidime/avibactam	64 mg/L (R)
Cefiderocol	0.12 mg/L (S)
Aztreonam	4 mg/L (I)
Imipenem	8 mg/L (R)
Meropenem	>8 mg/L (R)
Ciprofloxacin	>2 mg/L (R)
Amikacin	>32 mg/L (R)
Tobramycin	≤2 mg/L (S)
Colistin	≤2 mg/L (S)
Acquired resistance genes	*bla*_DIM-1_*, aphA1, aph(3'*)

^*a*^ I, susceptible, increased exposure.

**Fig 1:**
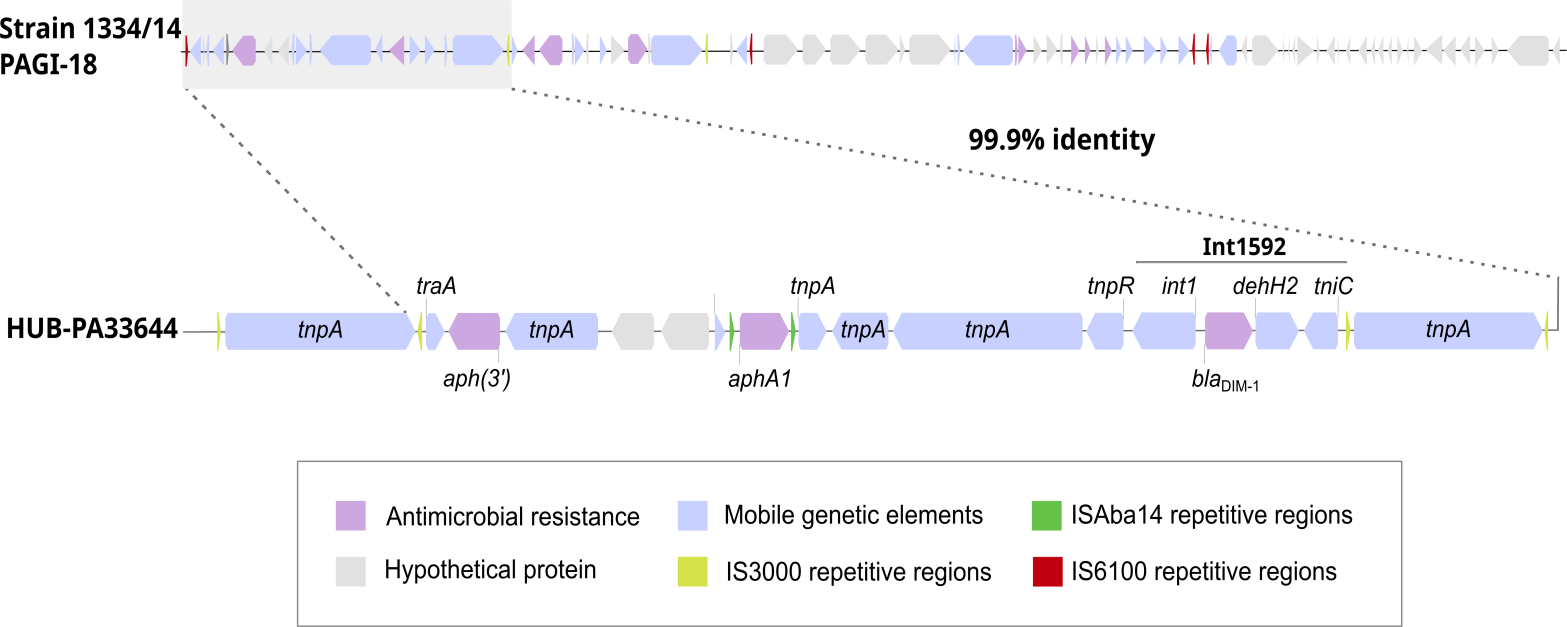
Representation of the genetic environment surrounding the integron Int1592 harboring *bla*_DIM-1_ in the strain HUB-PA33466 compared with the previously described PAGI-18 of strain 1334/14 (6).

The *bla*_DIM-1_ gene was located within a class 1 integron (In1592) containing an integrase type I gene (*intI),* as well as the *dehH2* gene coding for a dehalogenase enzyme and the *tniC* gene coding for a transposase. This integron has been reported previously in the Polish strain 1334/14 as part of a larger mobile genetic element known as the PAGI-18, a genomic island that contains multiple additional resistance determinants, repetitive elements, and transposition-related genes (6). Interestingly, the HUB-PA33644 strain carried the first fragment of the PAGI-18 genomic island, sharing 99.9% sequence identity with the corresponding region in the Polish strain (Fig. 1). However, the insertion sequences flanking this element differed between the two strains. In the original PAGI-18, the fragment was flanked by IS6100, whereas in our strain, the fragment was surrounded by IS3000 repeats. This suggests that In1592 may have been mobilized through different mechanisms in independent acquisition events, resulting in variations in its insertion site. In addition to the integron In1592 carrying the *bla*_DIM-1_ gene, the mobilizable element in HUB-PA33644 also encoded the aminoglycoside resistance genes *aph(3'*) and *aphA*. The remaining genes in the region were mostly associated with mobility, including multiple *tnpA* genes encoding transposases that mediate the movement of transposable elements, the *tnpR* gene encoding a resolvase involved in site-specific recombination, and the *traA* gene, encoding a conjugation protein. These genomic features underscore the high potential for horizontal transfer and integration of resistance elements within diverse bacterial hosts.

Taken together, the conserved integron structure flanked by insertion sequences and the presence of multiple mobility genes highlight the dynamic dissemination of resistance determinants in *P. aeruginosa*. The patient’s history of broad-spectrum antibiotic use prior to admission likely imposed a strong selective pressure that favored the persistence of this MDR strain and facilitated the maintenance of the rare *bla*_DIM-1_ carbapenemase gene. This genomic context, featuring associated transposases and conjugation genes, demonstrates how antimicrobial selective pressure can promote the proliferation of uncommon carbapenemase genes via mobile genetic elements. The detection of DIM-1 in Spain underscores the ongoing threat of MBL dissemination through mobile genetic elements, such as integrons and genomic islands. These findings emphasize the critical role of uncommon resistance genes and their associated mobile elements, reinforcing the need for continuous genomic surveillance and in-depth contextual characterization to monitor the evolution and mitigate their spread.

## References

[1] Manohar P, Babu S, Bozdogan B, Ramesh N. 2018. Identification of bla_DIM-1_ metallo-β-lactamase gene in Pseudomonas aeruginosa isolated from Tamil Nadu, India. J Glob Antimicrob Resist 13:7–8. doi:10.1016/j.jgar.2018.02.018 29501918

[2] Booth MPS, Kosmopoulou M, Poirel L, Nordmann P, Spencer J. 2015. Crystal structure of DIM-1, an acquired subclass B1 metallo-β-lactamase from Pseudomonas stutzeri. PLoS One 10:e0140059. doi:10.1371/journal.pone.0140059 26451836 PMC4599830

[3] Walsh TR, Toleman MA, Poirel L, Nordmann P. 2005. Metallo-β-lactamases: the quiet before the storm? Clin Microbiol Rev 18:306–325. doi:10.1128/CMR.18.2.306-325.2005 15831827 PMC1082798

[4] Poirel L, Rodríguez-Martínez JM, Al Naiemi N, Debets-Ossenkopp YJ, Nordmann P. 2010. Characterization of DIM-1, an integron-encoded metallo-β-lactamase from a Pseudomonas stutzeri clinical isolate in the Netherlands. Antimicrob Agents Chemother 54:2420–2424. doi:10.1128/AAC.01456-09 20308383 PMC2876379

[5] Deshpande LM, Jones RN, Woosley LN, Castanheira M. 2014. Retrospective molecular analysis of DIM-1 metallo-β-lactamase discovered in Pseudomonas stutzeri from India in 2000. Antimicrob Agents Chemother 58:596–598. doi:10.1128/AAC.01541-13 24145536 PMC3910751

[6] Urbanowicz P, Izdebski R, Baraniak A, Żabicka D, Ziółkowski G, Hryniewicz W, Gniadkowski M. 2019. Pseudomonas aeruginosa with NDM-1, DIM-1 and PME-1 β-lactamases, and RmtD3 16S rRNA methylase, encoded by new genomic islands. J Antimicrob Chemother 74:3117–3119. doi:10.1093/jac/dkz262 31211367

[7] Tada T, Hishinuma T, Watanabe S, Uchida H, Tohya M, Kuwahara-Arai K, Mya S, Zan KN, Kirikae T, Tin HH. 2019. Molecular characterization of multidrug-resistant Pseudomonas aeruginosa isolates in hospitals in Myanmar. Antimicrob Agents Chemother 63:e02397-18. doi:10.1128/AAC.02397-18 30803967 PMC6496111

[8] Tran HA, Vu TNB, Trinh ST, Tran DL, Pham HM, Ngo THH, Nguyen MT, Tran ND, Pham DT, Dang DA, Shibayama K, Suzuki M, Yoshida L-M, Trinh HS, Le VT, Vu PT, Luu TVN, Bañuls A-L, Trinh KL, Tran VA, Tran HH, van Doorn HR. 2021. Resistance mechanisms and genetic relatedness among carbapenem-resistant Pseudomonas aeruginosa isolates from three major hospitals in Hanoi, Vietnam (2011-15). JAC Antimicrob Resist 3:dlab103. doi:10.1093/jacamr/dlab103 34322671 PMC8313516

[9] Janice J, Agyepong N, Owusu-Ofori A, Govinden U, Essack SY, Samuelsen Ø, Sundsfjord A, Pedersen T. 2021. Carbapenem resistance determinants acquired through novel chromosomal integrations in extensively drug-resistant Pseudomonas aeruginosa. Antimicrob Agents Chemother 65:e0028921. doi:10.1128/AAC.00289-21 33941520 PMC8373256

[10] Mei L, Song Y, Liu D, Li Y, Liu L, Yu K, Jiang M, Wang D, Wei Q. 2023. Characterization of a mobilizable megaplasmid carrying multiple resistance genes from a clinical isolate of Pseudomonas aeruginosa. Front Microbiol 14:1293443. doi:10.3389/fmicb.2023.1293443 38088964 PMC10711205

[11] Sengeruan LP, Omar OS, Kanje LE, Kimu P, Wadugu B, van Zwetselaar M, Kumburu H, Sonda T, Chugulu S, Mshana J. 2025. Distribution of carbapenemase genes associated with global high-risk sequence types in Pseudomonas aeruginosa isolates from chronic leg ulcer patients in northern Tanzania. Int Wound J 22:e70735. doi:10.1111/iwj.70735 40708548 PMC12290302

[12] Del Barrio-Tofiño E, López-Causapé C, Oliver A. 2020. Pseudomonas aeruginosa epidemic high-risk clones and their association with horizontally-acquired β-lactamases: 2020 update. Int J Antimicrob Agents 56:106196. doi:10.1016/j.ijantimicag.2020.106196 33045347

